# Optimized Dyeing Process for Enhancing the Functionalities of Spent Coffee Dyed Wool Fabrics Using a Facile Extraction Process

**DOI:** 10.3390/polym11040574

**Published:** 2019-03-28

**Authors:** Jihyun Bae, Kyung Hwa Hong

**Affiliations:** 1 College of Human Ecology, Department of Clothing and Textiles, Hanyang University, Seoul 04763, South Korea; jbae2@hanyang.ac.kr; 2 College of Natural Science, Kongju National University, Chungnam 32588, South Korea

**Keywords:** wool, spent coffee, tannin, dyeing, antibacterial property, antioxidant property

## Abstract

Spent coffee grounds are the byproduct of coffee brewing and are generally discarded as waste. However, spent coffee has high levels of organic compounds that have multiple biological effects, including antibacterial and antioxidant activities. In this light, spent coffee grounds were tested for fabric dyeing to both functionalize as well as color the fabrics. The dyeing solution was prepared by extracting spent coffee grounds collected from a local coffee house by using a manual espresso machine. The spent coffee extract was applied to wool fabrics using a laboratory infrared dyeing machine. After the dyeing process was completed, the fabrics were mordanted with a tannic acid aqueous solution. To optimize the dyeing conditions, the times and temperatures during the process were varied, and the functionalities and other properties including color and strength of the wool fabrics dyed with the spent coffee extract were investigated. The wool fabrics dyed with the spent coffee extract were significantly colored, and the color withstands the effect of washing and light exposure. Moreover, the dyeing process with the spent coffee extract and the mordanting process with tannic acid gave the wool fabrics antibacterial and antioxidant properties.

## 1. Introduction

Approximately 7.4 million tons of coffee is produced each year and it is the most consumed luxury table beverage in the world. Coffee is also the second most traded commodity by volume after petroleum, indicating that the economic impact of coffee is substantial [1]. In 2017 in Korea alone, approximately 26.5 billion cups of coffee were consumed. Considering the population of Korea, approximately 512 cups of coffee were consumed per person in 2017. Korea’s coffee market exceeded 10 trillion KRW (ca. 8.76 billion USD) for the first time in 2017. This market size is more than three times greater than it was 10 years ago [2]. Since coffee consumption has increased so dramatically, the amount of spent coffee grounds produced after brewing coffee cannot be ignored. In contrast to the rapid growth of the coffee market, the development of efficient treatments for spent coffee grounds is difficult because there is no established system for collecting and recycling spent coffee grounds [3]. Spent coffee grounds are not just food garbage; they are also a major cause of environmental pollution since they produce a large amount of methane gas, which has an adverse effect on global warming. The effect of methane gas is assumed to be approximately 2.5 times worse than that of carbon dioxide, and the amount of carbon dioxide produced by spent coffee grounds is approximately 1.6 times more than that produced by black tea [4]. However, spent coffee grounds still contain many functional components, such as phenolic compounds, terpenes, caffeine, and Maillard reaction products. Therefore, treatment of this bio-waste by simply burying it in a landfill or incinerating it may be wasting a valuable resource.

Our group recently conducted a study on the dyeing and finishing of cotton and wool fabrics by using spent coffee extract prepared by the extraction method suggested by Mussatto et al. [5]. This method was reported to be effective for extracting active components from spent coffee grounds. It involves placing the spent coffee grounds in a 60% aqueous methanol solution and shaking the solution for 60 min in a water bath at 60 °C [6,7]. The study showed that the fabrics treated with the spent coffee extract displayed a good antioxidant capacity and significant antibacterial activity, especially towards Gram-positive bacteria. In particular, the spent coffee extract showed strong color fastness in the fibers containing amide groups in their polymers (such as those found in wool fiber). This strong color manifestation indicates that spent coffee grounds may be an effective material for fiber dyeing and functionalization. Recently, we found that higher concentrations of effective components such as total phenolics and total condensed tannins are present in the spent coffee extract prepared by a typical espresso machine than in the extract prepared via a methanolic extraction [5,6,7]. Thus, machine extraction may be a good extraction method for fiber dyeing because it does not require methanol, a toxic solvent, which would need to be evaporated from the extract for fabric applications such as dyeing. In the present study, spent coffee extract prepared by using an espresso machine was used for wool dyeing, and the optimal dyeing conditions were investigated. In addition, tannic acid was used as a mordant in the fabric to improve its functionalities and the coloring effect of the spent coffee extract on the wool fabric. In addition, tannic acid is well known as an eco-friendly mordant and many researchers have been using it for attaining high quality natural dyeing along with imparting multifunctional properties from textile dyes [8,9].

## 2. Materials and Methods 

### 2.1. Sample Material and Chemicals 

A commercially available scoured wool fabric (ISO 105-F01; plain woven 125 g/m2) was prepared (Testfabrics Inc. West Pittston, PA, USA). Spent coffee grounds (Coffea arabica L.) were supplied from a local coffee house in Gongju, Korea. Folin-Ciocalteu reagent, gallic acid (≥97%), vanillin (99%), (+)-catechin hydrate (≥98%), potassium iodate, sodium bicarbonate, formic acid, acetonitrile, trigonelline, protocatechuic acid, tannic acid (ACS reagent, Mw: 1701.20), chlorogenic acid, and caffeine were purchased from Sigma-Aldrich (St. Louis, MO, USA). Free radical DPPH (1,1-diphenyl-2-picrylhydrazyl) was purchased from Calbiochem (San Diego, CA, USA). All reagents were used as received without further purification. 

### 2.2. Extraction of Spent Coffee Grounds to Prepare the Dyeing Solution

A manual espresso machine (Gaggia Gran Prestige, Milano, Italy) at a pump pressure of 15 bar was utilized for extracting dying solution from the spent coffee. The extract was used as a stock solution for the dyeing of fabrics in this study. 

### 2.3. Dyeing Process

Wool fabrics were cut into 30 cm × 30 cm pieces, and each piece was immersed in a vessel containing the stock solution of the spent coffee extract (bath ratio = 1:30). A laboratory infrared (IR) dyeing machine (Daelim Starlet Co., Ltd; Gyeonggi-do, Korea) was used for the dyeing process. The temperature of the dyeing bath was gradually increased (ca. 3 °C/min) up to the designated temperature (60, 90, and 120 °C), and then that temperature was maintained while the vessels were rotated at 45 rpm for the designated dyeing time (30, 60, 90, and 120 min). The dyed wool fabrics were thoroughly rinsed with deionized water and squeezed using a padder to obtain 100 wt % of a specified wet pick-up rate. 

### 2.4. Mordanting Process

The wool fabrics dyed with the spent coffee extract were subsequently mordanted as follows. After being squeezed with a padder, the damp wool fabrics were put into vessels containing a 1 wt % tannic acid aqueous solution (bath ratio = 1:30). The vessels were then shaken at 130 rpm for 60 min at 85 °C. The wool fabrics were next thoroughly rinsed with deionized water and dried in a convection oven at 60 °C.

### 2.5. Analysis of the Spent Coffee Extract

The antioxidant compounds in the spent coffee extract were analyzed by high-performance liquid chromatography (HPLC) using a diode array detector (Agilent Technologies, 1260 Infinity, Waldbronn, Germany). A Kinetex 5-μm C18 column (150 mm × 4.6 mm i.d., Phenomenex, Torrance, CA, USA) was employed at 40 °C. The antioxidant compounds were separated by a gradient mobile phase consisting of (A) 0.1% formic acid and (B) acetonitrile at a flow rate of 1 mL/min. The gradient was programmed as follows: 0–10 min, 15%–37% B; 5–10 min, 37%–80% B; 10–12 min, 80%–100% B; and 12–13 min, 100%–15% B. The major antioxidant compounds were identified based on the similarities between their experimental retention times and ultraviolet-visible (UV-Vis) spectra and those of pure authentic standards (trigonelline, gallic acid, chlorogenic acid, and caffeine). The total phenolic content in the spent coffee extract was measured by the colorimetric method described by Singleton and Rossi [10]. Folin–Ciocalteu reagent (2.5 mL, previously diluted with water 1:10, v/v) and 2 mL of 75 g/L aqueous sodium carbonate were added to 0.5 mL of an aqueous solution of the extract. The mixture was kept at 50 °C for 5 min, and after cooling, the absorbance was measured at 760 nm (Biomate5 spectrophotometer, Thermo, Waltham, MA, USA). The total phenolic content was calculated in terms of gallic acid equivalents (GAE) from the calibration curve of gallic acid standard solutions (2–40 μg/mL) and is expressed as mg gallic acid equivalent (GAE)/mg of extract (on a dry basis). The total tannin content in the spent coffee extract was determined by the vanillin/HCl method described by Broadhust and Jones [11] with some modifications. An aliquot of the extracts (1 mL) was added to the vanillin reagent (2 mL), which had been prepared by dissolving vanillin in methanol (0.5%, w/v). Aqueous HCl (2 mL, 4%, v/v) was added to the mixture, and then the mixture was incubated in the dark. The absorbance was measured at 500 nm after 20 min of incubation. Different concentrations (500–3000 μg/mL) of (+)-catechin standards were used to calculate the condensed tannin content. The total tannin content was expressed as mg of tannic acid equivalents (TAE)/mL. All samples were analyzed in triplicate, and the mean value was calculated.

### 2.6. Characteristics of the Fabrics Dyed with the Spent Coffee Extract

Color properties, in terms of the L*, a*, and b* values, and color differences, ΔE, of the dyed fabrics were investigated using a spectrophotometer (CM-2500d, Konica Minolta, Inc., Osaka, Japan). The color strength (K/S) values were assessed using the Kubelka–Munk Equation (1):

(1) $$K/S=\frac{{\left(1-R\right)}^{2}}{R}$$

In the above equation, R is the decimal fraction of the reflectance of the dyed fabric.

Color fastness was investigated as follows: color fastness to washing (ISO 105 C06: 2010, A2S, 30 min mechanical wash at 40 ± 2 °C in 0.4% European Colorfastness Establishment (ECE) reference detergent and 0.1% sodium perborate tetrahydrate solution with 10 steel balls); color fastness to light (ISO 105 B02: 2014, Xenon-arc lamp, blue scale). All tests were conducted at least in triplicate for all samples.

The shrinkage rate of dyed fabrics was determined based on the fabric count values measured via fabric analyzing glass, and calculated using Equation (2):

(2) $$\mathrm{Shrinkage}\;\left(\%\right)\;=\;\frac{D-P}{D}\times 100$$

In the above equation, D and P represent the fabric count values of dyed fabrics and pristine fabrics, respectively.

The tensile strength of the fabrics was measured by the cut strip method (modified ASTM D5035) only in the weft direction using an Instron 5543 system (Norwood, MA, USA); 300 mm/min, gauge length: 50 mm, specimen width: 25 mm. 

Fourier transform infrared (FT–IR) spectrometry was performed using a Spectrum 100 Optica FT–IR instrument (PerkinElmer, Waltham, MA, USA) with a resolution of 4 cm^−1^. The FT–IR measurements were carried out using an attenuated total reflectance (ATR) technique.

The ability of the dyed fabrics to impede microbial growth and retention was tested using *Staphylococcus aureus* (ATCC 6538; a Gram-positive bacterium) and *Klebsiella pneumoniae* (ATCC 4352; a Gram-negative bacterium) cultures according to an established protocol (KS K 0693).

(3) $$\mathrm{Reduction}\;\mathrm{of}\;\mathrm{bacteria}\;\left(\%\right)=\frac{\left(B-A\right)}{B}\times 100$$

In the above equation, A and B represent the surviving bacterial cells (colony-forming units mL-1) on the plates inoculated with the bacterial solution derived from the dyed fabric and a control solution derived from untreated fabric, respectively.

The antioxidant activity of the dyed fabrics was measured with DPPH using a previously reported method [12]. More details are presented in our previous papers [6,7]. Lower absorbances of the solutions indicated higher DPPH scavenging abilities. The DPPH scavenging ability was calculated using Equation (4).

(4) $$\mathrm{DPPH}\;\cdot \;\mathrm{scavenging}\;\mathrm{activity}\;\left(\%\right)\;=\;\frac{C-S}{C}\;\times \;100$$

In the above equation, S and C represent the absorbance at 517 nm of the sample from the dyed fabric and that of the control from the untreated fabric, respectively.

## 3. Results

### 3.1. Analysis of the Components of the Spent Coffee Extract

**Figure 1:**
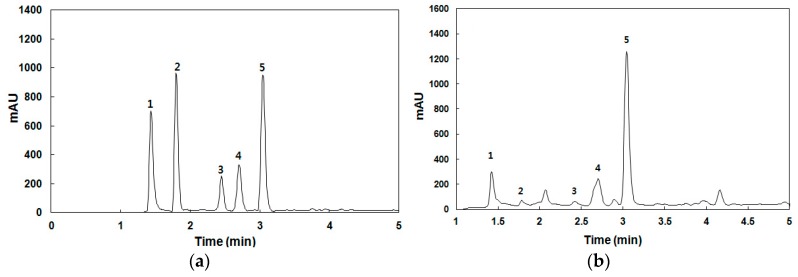
High-performance liquid chromatograms of standards (**a**) and spent coffee extract (**b**) at 258 nm. 1: trigonelline; 2: gallic acid; 3: protocatechuic acid; 4: chlorogenic acid; and 5: caffeine.

Four compounds were identified in the spent coffee extract by comparing their retention times and UV-Vis spectra with those of authentic standards (pure chemicals). Although a tiny peak appeared near the retention time corresponding to protocatechuic acid in Figure 1b, its UV-Vis spectrum was not identical with that of protocatechuic acid. This revealed that there is no protocatechuic acid in the spent coffee extract prepared in this research, even though the functional compound is known to exist in coffee extract [13]. The other four identified peaks were phenolic compounds (gallic acid and chlorogenic acid) and nitrogenous compounds (trigonelline and caffeine). Among the compounds identified, caffeine was the most abundant in the spent coffee extract. These results are consistent with previous studies [6]. The total phenolic content and total tannin content in the spent coffee extract were 2.63 g/L(GAE) and 10.20 g/L(TAE), respectively. For comparison, the total phenolic and total tannin content in the spent coffee extract prepared using methanolic method were 2.00 g/L(GAE) and 0.61 g/L(TAE), respectively, according to our previous study [6,7]. Therefore, it was found that all the functional compounds extracted by the espresso machine are present in much greater quantities than those extracted by the methanolic method. 

### 3.2. Fourier Transform Infrared (FT–IR) Spectra of the Wool Fabrics Dyed with the Spent Coffee Extract

**Figure 2:**
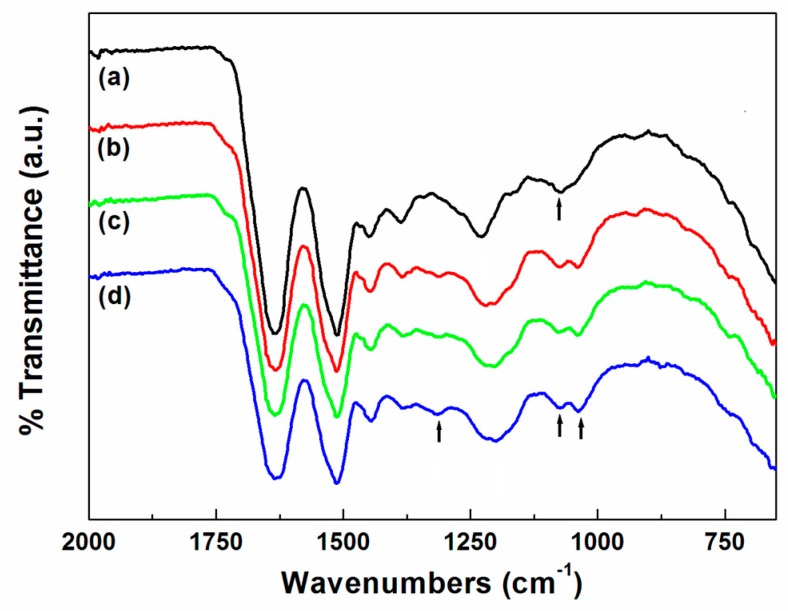
Fourier transform infrared-attenuated total reflectance (FT–IR-ATR) spectra of wool fabrics dyed with spent coffee extract for 60 min as a function of dyeing temperature (all dying samples were mordanted with 1 wt % tannic acid aqueous solution): (**a**) untreated, (**b**) 60 °C, (**c**) 90 °C, and (**d**) 120 °C.

**Figure 3:**
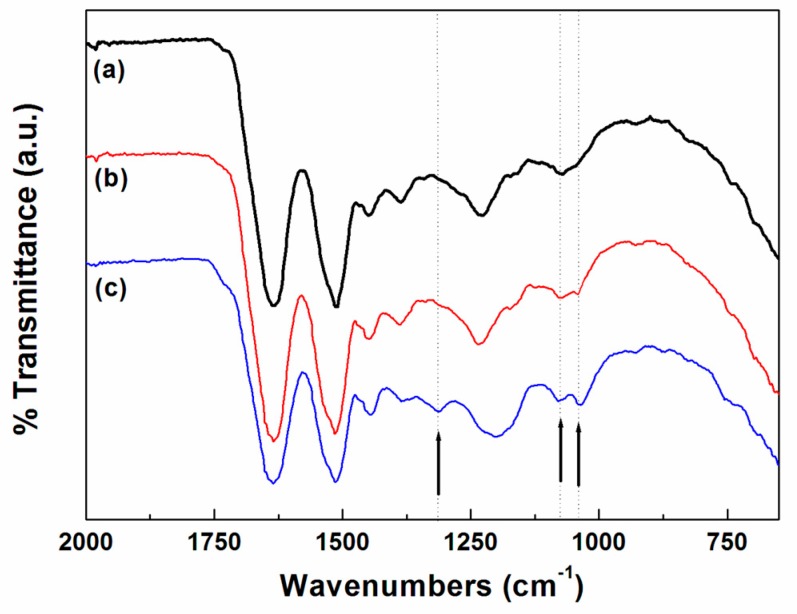
FT–IR-ATR spectra of wool fabrics dyed with spent coffee extract at 90 °C for 60 min: (**a**) untreated, (**b**) not mordanted, and (**c**) mordanted with 1 wt % tannic acid.

The FT–IR spectra of the wool fabrics dyed with the spent coffee extract and mordanted with tannic acid are shown in Figure 2. Wool fabrics are made from protein fibers containing various functional groups, such as carboxyl (–COOH), amino (–NH_2_), and hydroxyl (–OH) groups [14]. Thus, all wool fibers displayed similar absorption bands at 3283 cm^−1^ (N–H and O–H), 2873 cm^−1^ (–CH_2_), 1634 cm^−1^ (amide I), 1512 cm^−1^ (amide II), and 1229 cm^−1^ (amide III). However, new peaks at 1312 cm^−1^ and 1037 cm^−1^ were observed in the spectra of the wool fabrics dyed with the spent coffee extract and mordanted with tannic acid. These bands are presumed to be driven by the C–O stretching of the ester attributed to tannin in the wool fabrics dyed with spent coffee extract and mordanted with tannic acid. This is because the new bands were more intense after mordanting with tannic acid, as shown in Figure 3. Tannin is very soluble in water, and the hydrolyzable tannins break down via hydrolysis to give gallic acid, a type of phenolic compound [15,16,17]. Therefore, a significant amount of phenolic compounds was chemically attached to the wool fibers through the mordanting process as well.

### 3.3. Apparent Colors of the Wool Fabrics Dyed with the Spent Coffee Extract

**Figure 4:**
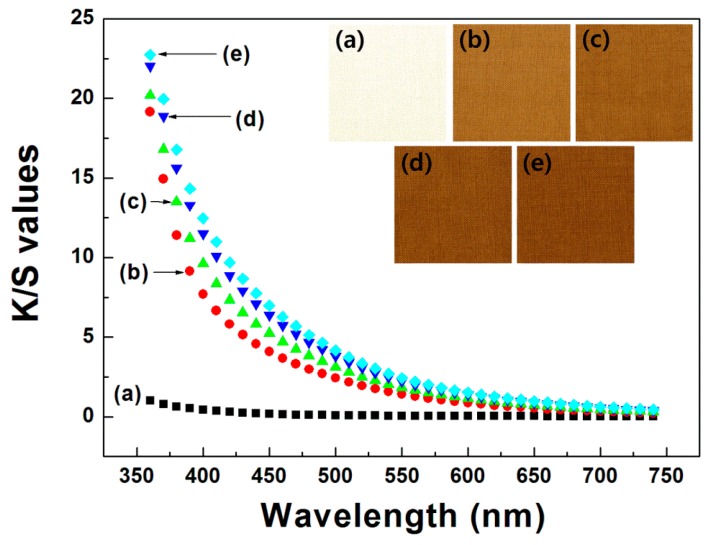
Color strength (K/S) values of wool fabrics dyed with spent coffee extract at 90 °C as a function of dyeing time (all dying samples were mordanted with 1 wt % tannic acid aqueous solution): (**a**) untreated, (**b**) 30 min, (**c**) 60 min, (**d**) 90 min, and (**e**) 120 min.

**Figure 5:**
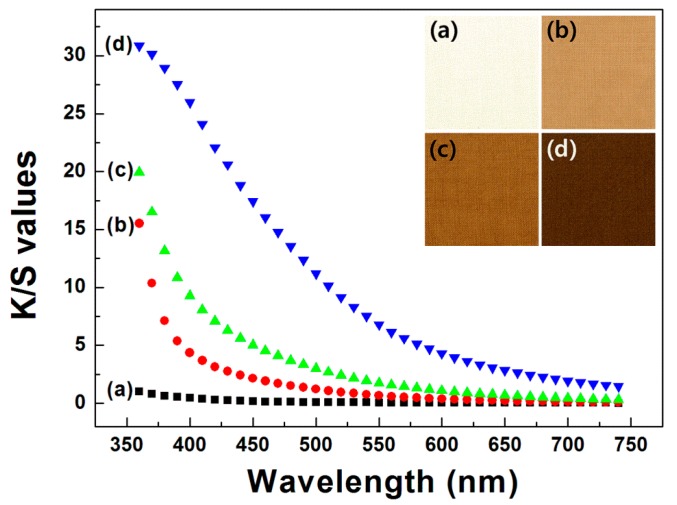
K/S values of wool fabrics dyed with spent coffee extract for 60 min as a function of dyeing temperature (all dying samples were mordanted with 1 wt % tannic acid aqueous solution): (**a**) untreated, (**b**) 60 °C, (**c**) 90 °C, and (**d**) 120 °C.

Dyeabilities of the wool fabrics dyed with the spent coffee extract and mordanted with tannic acid were improved overall as the dyeing time and dyeing temperature increased. In particular, dyeing temperature had a significant effect on the dyeability, while dyeing time negligibly did, as shown in Figure 4 and Figure 5. In addition, the wool fabrics dyed with the spent coffee extract and mordanted with tannic acid showed increasing a* values but decreasing b* and L* values as the dyeing time and dyeing temperature increased, as shown in Table 1. This result indicates that reddish hue became dominant but yellowish hue became faint in the wool fabrics as the dyeing time and dyeing temperature increased. It appears that the brown pigments were primarily caused by melanoidins produced during the process of roasting coffee beans. Melanoidins are brown compounds and are known to have several biological activities, such as antioxidant, antimicrobial, anticarcinogenic, anti-inflammatory, antihypertensive, and antiglycative activities. However, knowledge of melanoidins in coffee including the chemical structures is still lacking [18]. On the other hand, it was observed that mordanting with 1 wt % tannic acid has little effect on the color appearance of wool fabrics dyed with spent coffee extract, as shown in Table 1. To identify the levelness of wool fabrics dyed with the spent coffee extract, we investigated the color difference of two different points with at least 10 cm distance within each sample. Consequently, any unlevelness was not noticed in the wool fabrics dyed with the spent coffee extract.

Considering the color fastness of the wool fabrics dyed with the spent coffee extract and mordanted with tannic acid, the wool fabrics showed a high level of color fastness to washing (4.5 grade); in contrast, they exhibited somewhat inferior color fastness to light (3–4 grade), as shown in Table 2. However, it was found that the levels of color fastness of the wool fabrics dyed with the spent coffee extract and mordanted with tannic acid may be higher overall than those of fabrics dyed with other natural pigments [19,20].

### 3.4. Mechanical Properties of the Wool Fabrics Dyed with the Spent Coffee Extract

Shrinkage of the wool fabrics occurred through the dyeing and mordanting process, as shown in Table 3. The shrinkage became intensified as the dyeing condition became progressively harsh, i.e. higher temperature and longer time of the dyeing process. In particular, significant shrinkage of the wool fabrics was induced by high-temperature dyeing. The mechanical strength of the wool fabrics dyed with the spent coffee extract and mordanted with tannic acid also depreciated drastically after the high-temperature dyeing at 120 °C. Considering the color appearance and mechanical properties of the wool fabrics dyed with the spent coffee extract, it was discovered that the dyeing process at 90 °C for 90 min would be optimal conditions for wool fabrics. 

### 3.5. Functional Properties of the Wool Fabrics Dyed with the Spent Coffee Extract 

Table 4 and Table 5 show the antibacterial activities of the wool fabrics dyed with the spent coffee extract and mordanted with tannic acid. Overall, they showed significant antibacterial activities against *K. pneumoniae*, a Gram-negative bacterium, as well as against *S. aureus*, a Gram-positive bacterium. The effects of dyeing time and dyeing temperature were not significant. However, the wool fabric dyed with spent coffee extract but without mordanting shows insufficient antibacterial ability particularly to *K. pneumoniae*, as shown in Table 4 (dyeing time 90*). Therefore, it was found that the antibacterial activities of the wool fabrics dyed with the spent coffee extract were dramatically enhanced by mordanting with tannic acid following the dyeing process.

Table 6 and Table 7 show the antioxidant activities of the wool fabrics dyed with the spent coffee extract and mordanted with tannic acid. All the wool fabrics dyed with the spent coffee extract and mordanted with tannic acid showed antioxidant capacity greater than 93%, and the activities were increased by a small amount with increased dyeing temperature (Table 7). However, it was observed that the antioxidant capacity is also primarily attributed to mordanting. This is because a significant amount of phenolic compounds were attached to the wool fibers via the mordanting process. Phenolic compounds are found in many plants and are known to possess diverse health-promoting effects such as antimelanogenic, antioxidant, antineoplastic, and bacteriostatic properties [21,22,23]. It was reported that chlorogenic acid is the major phenolic component in spent coffee extract, and gallic acid might be abundant in the mordanting solution. Additionally, the non-phenolic compounds in coffee extracts, such as caffeine and melanoidins, can also contribute to the antioxidant activity and scavenging of hydroxyl radicals (a type of highly active reactive oxygen species (ROS)) [24].

## 4. Conclusions

Wool fabrics were dyed with spent coffee extract and mordanted with tannic acid to generate functionalized and colored textiles and to recycle spent coffee grounds, which are a major component of bio-waste. The spent coffee extract was simply prepared by using a manual espresso machine, and the extract contained many functional compounds, including phenolic compounds (gallic acid and chlorogenic acid) and nitrogenous compounds (trigonelline and caffeine). The spent coffee extract significantly colored wool fabrics brown via the dyeing process, and the coloring effect was enhanced by increasing the dyeing time and temperature. However, dyeing at extremely high temperature was observed to deteriorate the mechanical strength of wool fabrics and, therefore, dyeing at 90 °C for 90 min would be the optimal condition for dyeing wool fabrics with spent coffee extract. On the other hand, dyeing with the spent coffee extract alone imparted a limited level of functionality to the fabrics, such as antibacterial activity and antioxidant capacity. However, mordanting with tannic acid after the dyeing process could enhance the functionalities of the wool fabrics dyed with spent coffee extract and improve the color fastness of the fabrics to light.

## Figures and Tables

**Table 1:** Color values of wool fabrics dyed with spent coffee extract and mordanted with 1 wt % tannic acid aqueous solution.

Dyeing Conditions		L*	a*	b*	ΔE
Temperature (°C)	Time (min)				
Untreated		86.54	−0.59	12.68	-
Only mordanted		85.02	1.04	12.96	2.24
90	30	54.12	9.59	27.40	37.03
	60	50.29	9.82	26.88	40.30
	90	47.40	10.12	26.62	42.91
	120	46.08	10.19	26.49	44.09
60	60	64.50	8.72	28.64	28.76
90		50.88	9.74	26.96	39.77
120		31.38	9.77	20.78	56.71

**Table 2:** Color fastness of wool fabrics dyed with spent coffee extract and mordanted with 1 wt % tannic acid aqueous solution.

Dyeing Conditions		Color Change to Washing (Grade)	Color Change to Light (Grade)
Temperature (°C)	Time (min)		
90	30	4-5	3
	60	4-5	3-4
	90	4-5	3-4
	120	4-5	3-4
60	60	4-5	2-3
90		4-5	3-4
120		4-5	4

**Table 3:** Shrinkage and tensile strength of wool fabrics dyed with spent coffee extract and mordanted with 1 wt % tannic acid aqueous solution.

Dyeing Conditions		Warp	Weft	Fabric Count	Shrinkage (%)	Tensile Strength (N)
Temperature (°C)	Time (min)					
Untreated		60	48	2880	0	117
Only mordanted		60	49	2940	2.04	116
90	30	62	50	3100	7.10	122
	60	63	50	3150	8.57	123
	90	63	50	3150	8.57	126
	120	62	51	3162	8.92	123
60	60	61	50	3050	5.57	125
90		63	50	3150	8.57	123
120		73	61	4453	35.32	76

**Table 4:** Antibacterial activity of wool fabrics dyed with spent coffee extract at 90 °C as a function of dyeing time (all dying samples were mordanted with 1 wt % tannic acid aqueous solution except 90* sample).

Dyeing Time (min)	Reduction % of *S. aureus*	Reduction % of *K. pneumoniae*
Pristine wool	28.2	32.5
30	99.2	93.1
60	99.7	97.5
90	99.7	99.6
90 *	76.5	43.6
120	99.6	96.2

* Wool fabrics dyed with spent coffee extract but not mordanted.

**Table 5:** Antibacterial capacity of wool fabrics dyed with spent coffee extract for 60 min as a function of dyeing temperature (all dying samples were mordanted with 1 wt % tannic acid aqueous solution).

Dyeing Temperature (°C)	Reduction % of *S. aureus*	Reduction % of *K. pneumoniae*
Pristine wool	28.2	32.5
60	99.6	94.1
90	99.7	97.5
120	99.9	99.2

**Table 6:** Antioxidant activity of wool fabrics dyed with spent coffee extract at 90 °C as a function of dyeing time (all dying samples were mordanted with 1 wt % tannic acid aqueous solution except 90* sample).

Dyeing Time (min)	DPPH Scavenging Activity (%)
Pristine Wool	59.65
30	94.28
60	94.46
90	94.83
90 *	59.65
120	94.68

* Wool fabrics dyed with spent coffee extract but not mordanted.

**Table 7:** Antioxidant ability of wool fabrics dyed with spent coffee extract for 60 min as a function of dyeing temperature (all dying samples were mordanted with 1 wt % tannic acid aqueous solution).

Dyeing Temperature (°C)	DPPH Scavenging Activity (%)
Pristine wool	59.65
60	93.59
90	94.30
120	95.13
